# Microbial landscape of cooked meat products: evaluating quality and safety in vacuum-packaged sausages using culture-dependent and culture-independent methods over 1 year in a sustainable food chain

**DOI:** 10.3389/fmicb.2024.1457819

**Published:** 2024-09-12

**Authors:** Wilson José Fernandes Lemos Junior, Lucas Marques Costa, Carlos Alberto Guerra, Vanessa Sales de Oliveira, Angela Gava Barreto, Fabiano Alves de Oliveira, Breno Pereira de Paula, Erick Almeida Esmerino, Viviana Corich, Alessio Giacomini, André Fioravante Guerra

**Affiliations:** ^1^Department of Biology, University of Padova, Padova, Italy; ^2^BRC Ingredientes Ltda., Rio Claro, São Paulo, Brazil; ^3^Universidade Federal Rural do Rio de Janeiro (UFRRJ), Seropédica, Rio de Janeiro, Brazil; ^4^Centro Federal de Educação Tecnológica Celso Suckow da Fonseca (CEFET/RJ), Valença, Rio de Janeiro, Brazil; ^5^Department of Agronomy Food Natural Resources Animals and Environment (DAFNAE), University of Padova, Legnaro, Padua, Italy; ^6^Department of Land, Environment, Agriculture and Forestry—TeSAF Legnaro, Padova, Italy

**Keywords:** sausage microbiome, sustainable production, quality methods, cooked meat, meat

## Abstract

Over the last few decades, advancements in process safety and quality methods have been significantly improved, yet new challenges continue to emerge in the sustainable food supply chain. This study aimed to investigate some physicochemical and microbiological parameters impacting meat products, particularly cooked sausages, within a sustainable supply chain, focusing on quality, spoilage populations, and syneresis formation under vacuum conditions. A comprehensive analysis was conducted on 355 samples collected over four seasons using high-throughput sequencing (16S/ITS) and microbiological and physicochemical [pH and water activity (a_w_)] assessments. The microbial growth predictor MicroLab_ShelfLife was employed, and multiple factor analysis (MFA) and agglomerative hierarchical clustering (AHC) were utilized to understand how these variables influence the microbiome resilience of these products. Lactic and acetic acids were correlated with the microbiome of the sausages and the liquid coating covering them using metagenomic analyses. The study highlighted that 52% of the evaluated meat industries in southeastern Brazil are implementing effective protocols for sustainable chain production. The results indicated that the durability of vacuum-packaged cooked sausages was primarily influenced by storage temperature (RV coefficient of 0.906), initial microbial load (0.755), and a_w_ (0.624). Average microbial counts were 4.30 log cfu/g (initial), 4.61 (7°C/4 days), 4.90 (7°C/8 days), 6.06 (36°C/4 days), and 6.79 (36°C/8 days). Seasonal durability analysis revealed that winter had the highest average durability of 45.58 days, while summer had the lowest at 26.33 days. Yeast populations, including *Trichosporon* sp. and *Candida* sp., were identified as key genera influencing spoilage dynamics. In addition, *Bacillus* species emerged as dominant spoilage microorganisms, highlighting the need for new critical controls. This study demonstrates the impact of metagenomic approaches, including *ITS* and *16S* amplicon sequencing, in revealing microbial community dynamics, storage temperature, and a_w_, which are essential for developing targeted interventions to enhance food safety and quality sustainably.

## Highlights

- The study emphasized the necessity of sustainable production practices, highlighting that 52% of the evaluated meat industries in southeastern Brazil are implementing effective protocols for sustainable chain production.
- An a_w_ value of 0.624 highlighted the importance of these factors in maintaining product quality.
- The study emphasized *Bacillus* sp. as a significant spoilage microorganism in the evaluated sausages. The study seasons, such as summer, significantly impact the spoilage rates of the vacuum-packaged cooked sausages.
- Syneresis formation under vacuum conditions was identified as a critical factor affecting sausage quality.

## 1 Introduction

Food loss and waste pose significant challenges across the entire food supply chain, contributing to environmental degradation, economic losses, and heightened food insecurity. New evaluations and updates in food production may become necessary due to shifts that align with new challenges related to food safety and quality, while respecting the sustainable new consumption profile and production (Kumar et al., 2023).

The meat supply chain has increasingly become a prominent research focus, with various studies exploring different facets of this complex system (Kharola et al., 2022; Caccialanza et al., 2023; Kumar et al., 2023). Additionally, there has been significant attention on consumer behavior and awareness, particularly examining the growing perception of meat products as having a substantial impact on climate change (Jansson et al., 2017). These changes can be extended to the consequences reflected in the profile of spoilage in this type of product during storage.

Among meat products, sausages are among the most produced, playing a crucial role in the economy of several countries worldwide (Lonergan et al., 2019). Cooked pork sausages are heat-processed products made from ground pork meat, fat, water and/or ice, emulsifiers, spices, flavors, and food additives, such as food preservatives and phosphates. They are stuffed in a natural or artificial casing and then cooked, ensuring that the minimum temperature reaches 72°C in the cold spot (Knipe, 2014). Regarding the packaging used, sausages are usually packaged and made available for sale in vacuum-sealed packages. In addition, the food industry, in response to market demand, generally establishes an expiration date of 2 months.

The natural microbiota of meat contains a variety of microorganisms, including Gram-positive and Gram-negative bacteria, molds, and yeasts. During sausage production, the cooking process applied to achieve 72°C in the cold spot can eliminate vegetative cells, excluding spore-forming groups, such as clostridia (James and James, 2014). Regulatory agencies recommend adding nitrate and nitrite-containing curing salt to achieve the typical reddish color and to prevent the growth of vegetative cells of *Clostridium botulinum*.

For several years, *C. botulinum* has been regarded as the most dreaded pathogen in this type of product. The trend of lowering the nitrite content and replacing raw ingredients with a new microbiome profile has altered the landscape of food quality and safety. Consequently, new issues have arisen during the production or storage processes. To enhance product quality and address the impact on human health within a sustainable supply chain, omics analyses serve as potent tools for identifying the origins of these emerging problems (Ferrocino et al., 2018).

Based on new trends, there is evidence that some microorganisms, such as sporous-forming *Bacillus*, can withstand the addition of curing salt (Majou and Christieans, 2018). To minimize the initial microbial load in the product, producers should ensure that post-cooking contamination is avoided, particularly from lactic acid bacteria (LAB), and manage the levels of *Bacillus* sp. to the lowest feasible extent (Iulietto et al., 2015).

Focusing on storage, new parameters need to be considered, such as the synergistic reactions in vacuum packaging and the growth of ropy slime-forming bacteria (e.g., *Bacillus* sp.), which, when combined, can be responsible for common defects in vacuum-packaged meat products. Thus, the visual aspect created coupled with typical color decaying may impact consumer appraisals and lead to the rejection of the product before the expiry date prescribed by the manufacturer (de Lima et al., 2022). Approximately one million tons (in carcass weight equivalent) of meat and meat products are lost annually (FAO, 2021). Therefore, based on the Food and Agriculture Organization (FAO) recommendation of meat consumption per day (71 g per healthy adult), this above-mentioned amount of meat is sufficient to feed ~14 million people per year. The hot seasons, such as summer, are considered critical for these losses. In addition, vacuum-packaged cooked sausages are traditionally sold at room temperature in several countries, including Brazil. Therefore, the manufacturers must collect spoiled products from the market and discard them, contributing to economic losses, food waste, and damages to the product brand (Ishangulyyev et al., 2019).

During sausage storage, the phenomenon of syneresis can play a crucial role. Syneresis refers to the expulsion of moisture or liquid from the sausage matrix, leading to the formation of visible droplets or a pool of liquid. This process is influenced by various factors, including temperature fluctuations, water activity, and the composition of sausages. It is important to highlight that the change for a sustainable supply chain can also interfere with this physico-chemical process. In certain cases, syneresis can be associated with undesirable changes in the texture, flavor, and the overall quality of sausages. Effective control measures during storage, such as maintaining consistent temperature and humidity levels, are vital to minimize syneresis and preserve the sensory attributes of sausages over time. Understanding and managing syneresis is essential for ensuring the optimal quality and consumer acceptance of stored sausages.

Reportedly, Food industries incorporate chemical preservatives and alter the sausage recipe or employ more stringent parameters during the cooking process to achieve enhanced microbial and chemical stability. Producers may also apply post-package chemicals and physical treatments to eliminate potential problems to interfere with the quality or safety aspects of the meat product (Barcenilla et al., 2022). However, from a sustainable perspective, these alternatives may be deemed undesirable due to their potential environmental and human health impacts. Therefore, it is necessary to understand the impact of these new alternatives on inhibiting microbial growth and heat-resistant groups, such as spore-forming bacteria, which can remain in the product (Morales et al., 2020). Therefore, a modest improvement in the durability of sausages is achieved beyond increasing the recipe cost following a sustainable supply chain. Initial microbial load, storage temperature, and a_w_ are among the critical factors that impact microbial growth in meat products. Moreover, they represent parameters that can be manipulated by food processing to improve the durability of vacuum-packaged cooked sausages (Korkeala and Björkroth, 1997).

Although the role of these factors in the spoilage process of vacuum-packaged cooked sausages is known, the level of relevance of each factor remains to be elucidated and updated for providing a new profile of spoilage from advanced methods to reduce biological and chemical risks. Therefore, this type of information is particularly useful to guide producers who aim to design strategies to increase the durability of these products in the markets. Thus, proper control of these parameters is mandatory to effectively inhibit microbial growth and achieve improvements in retaining the original sensory attributes of the product (Li et al., 2022).

Researching the microbiome is essential for promoting sustainable systems and enhancing the quality of meat. The diverse microbial communities in meat products significantly affect their safety, shelf life, and taste. The use of advanced technologies to study these microbiomes allows scientists to pinpoint which ones cause spoilage and find ways to control them without relying heavily on chemical preservatives. The approach not only helps in extending the shelf life of meat but also supports environmentally friendly production practices, reducing food waste and improving product quality. For example, understanding how specific bacteria, such as *Bacillus* species, spoil meat can lead to better storage and handling methods, ensuring longer-lasting and safer products (Ferrocino et al., 2018; Chen et al., 2020). Integrating microbiome research with sustainable practices not only benefits the food industry but also positively impacts the environment and overall food security (Cauchie et al., 2020; Caccialanza et al., 2023).

Against this background, this study aimed to investigate the impact of new factors contributing to the spoilage of vacuum-packaged cooked sausages, taking into consideration storage temperature and season parameters, through a prediction model, culturing, and omics approaches.

## 2 Materials and methods

### 2.1 Manufacturing of vacuum-packaged cooked sausages

In this study, the manufacturing process of sausages elucidated sustainable production practices and the substantial reduction of chemical preservatives.

### 2.2 Screening

The screening was carried out based on initial sampling, totaling 115 packages (*n* = 5 packages per group, a total of 23 industries) for a microbiological trial and 69 packages (*n* = 3 packages per group). The industries are located in the southeastern region of Brazil.

The production process for the meat products followed standard procedures, which included the following steps: input of raw materials, defrosting or breaking using a frozen block crusher, grinding with an industrial grinder (8–12 mm diameter plate), mixing with added food ingredients, such as lean meat, pork fat, spices, and filtrated water, stuffing in a 15 and 250 mm inner (diameter x length) natural pork casing, cooking to achieve 72°C in the coldest point of the sausage, cooling by immersion in a cold water bath, and packing in 16 × 25 cm vacuum pouches of polyamide and polyethylene using a vacuum-package system with two pieces of sausages per package. The samples were packed in boxes with reusable ice bricks and sent to a microbiology laboratory (Cefet/RJ, Valença, Brazil) immediately after manufacturing in isothermal trucks equipped with cold temperature control to preserve the initial microbial load. The samples were coded by the order in which they were received in the laboratory. The sample groups with an initial microbial load below 6.0 log colony-forming units per gram (cfu/g) were included in the study.

### 2.3 Culturing validation

To determine the initial microbial load in the sausage samples, plate count agar (PCA) was used for the total bacterial count, and de Man Rogosa Sharpe (MRS) agar was used as an internal control for the quantification of LAB. The procedure began by homogenizing 25 g of each of the five sausage samples in 225 mL of sterile buffered peptone water, creating a 1:10 dilution. The serial dilutions of the homogenized samples were prepared, and aliquots from these dilutions were spread-plated onto both PCA and MRS agar plates (HiMedia, Mumbai, India) in triplicate.

The plates were incubated at 37°C for 48 h. Following incubation, colonies were counted on the plates, which contained 30–300 colonies, which is considered an optimal range for accurate enumeration. The microbial load was calculated and expressed as colony-forming units per gram (cfu/g) of the samples. The total bacterial counts were determined using PCA, and LAB quantification was performed using MRS agar, following standard methodologies (Giraffa and Neviani, 2001).

### 2.4 Measurements of a_*w*_ and pH

An AquaLab LITE device (Decagon, Washington, USA), provided with a dielectric humidity sensor and infrared sample surface temperature, was utilized for a_w_ measurements. Before measurement, the equipment was calibrated using two standard solutions: K_2_SO_4_ (a_w_ 0.973, CAS 7778–80–5) and KCl (a_w_ 0.843, CAS 7447–40–7) provided by the manufacturer. The calibration ensured an accuracy of ±0.005. To obtain a uniform sample, a sausage piece was ground with an electric meat grinder (Centrífuga 1000, Britânia, Brazil). Over-grinding was avoided to prevent heating and potential measurement alterations. The sample was promptly taken to minimize humidity exposure. Approximately 7 mL of the sample was placed in a dish, ensuring it was filled without empty space at the bottom. Measurement stability was monitored using standard solutions during the analytical series. A waiting period of ~15 min was observed between each measurement after opening the equipment lid, and the procedure was performed in triplicate.

Non-destructive measurement of pH was performed using a portable meat pH meter device (pH-1900, Instrutherm, Brazil), equipped with a knife probe electrode (EPC-50, Instrutherm, Brazil) and automatic compensation of temperature. The sausages were randomly withdrawn from the packages, and the pH value was determined by directly sticking the electrode in five different positions of the sausages, including the ends and the central section of the pieces. Before measuring, the equipment was calibrated with buffer solutions, pH 4.00 and pH 6.88 at 20°C. A maximum error of ± 0.01 was considered accurate. The procedure was performed in triplicate.

### 2.5 Season and storage temperature evaluation

The samples for the next study were selected based on a criterion determined based on the initial microbial load. The microbial growth predictor MicroLab_ShelfLife reportedly loses sensitivity for samples with populations close to deceleration or stationary phases (Guerra et al., 2023). Thus, for more accuracy, new sampling was performed based on the criteria above from June 2021 to July 2022, covering the four seasons, N = 4 x 12 samples with five replicates. Only those samples that complied with the acceptance criterion considered in this study were selected. The microbial growth predictor named MicroLab_ShelfLife was used to perform the durability study and to evaluate the influence of initial microbial load, temperature fluctuation, and a_w_ on the durability of the sausages. One package per group was analyzed soon after being received in the laboratory to count the initial microbial load (time zero). The growth of natural microbiota in the packages was stimulated by conducting pair incubation of the samples at lower (7°C) and higher (36°C) temperatures. BOD incubators were used for precise temperature control. On the 4th day of the incubation, one package incubated at 4°C and another package incubated at 36°C were withdrawn to determine the total number of microorganisms. This procedure was repeated on day 8. A pour-plate method was carried out to determine the total number of viable bacteria, and the results (cfu/g) were calculated using at least two successive dilution levels, according to Equation 1.


(1) $$\begin{array}{l} N=\frac{\sum C}{V\;\left[n1+0.1n2\right)\;d} \end{array}$$

where ∑*C* corresponds to the colonies counted on the two plates retained from two successive dilutions (at least one of which contained a minimum of 10 colonies); *V* corresponds to the volume of the inoculum placed in each well (mL); *n1* and *n2* correspond to the number of wells selected in the first dilution and the number of wells selected in the second dilution, respectively; and *d* corresponds to the level of the first dilution retained.

**Figure 1:**
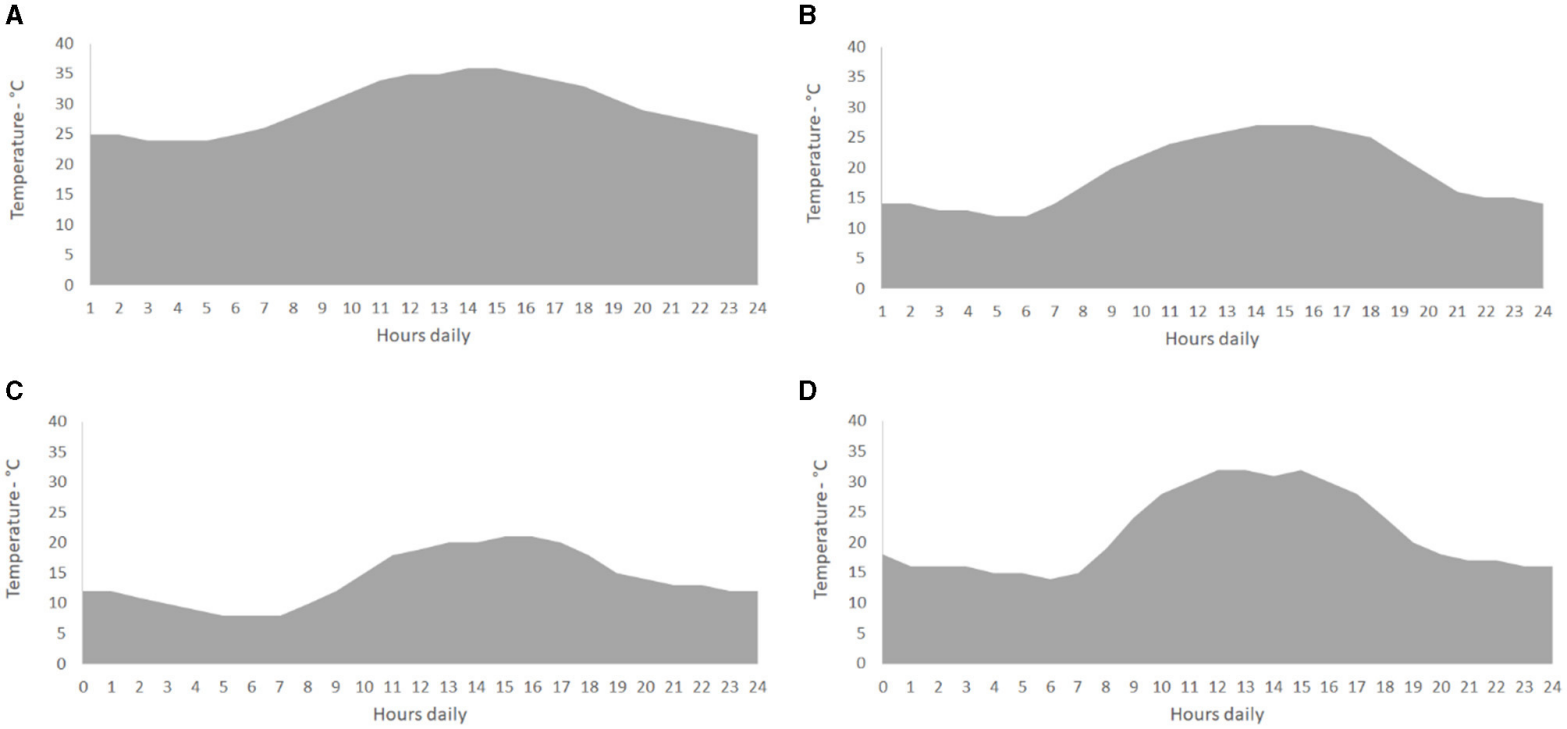
Temperature profile based on hourly variations during a 1-day period to represent the seasons: summer **(A)**, autumn **(B)**, winter **(C)**, and spring **(D)**. They were determined based on the measurements of AccuWeather (www.accuweather.com) for 2022. Latitude and longitude coordinates −22,246 and −43.7031; 22° 14′ 46″ South, 43° 42′ 11″ West (Valença, Rio de Janeiro, Brazil)—humid subtropical climate (Caf; Köppen-Geiger; Peel et al., 2007).

The results related to the colony counting were entered in the computational predictive modeling package to obtain information about the parameters of the microbial growth curve at a chosen dynamic temperature profile according to the AccuWeather forecast for 2022. Latitude and longitude coordinates (−22.246, 22° 14′ 46″ South; −43.7031, 43° 42′ 11″ West; Valença, Rio de Janeiro, Brazil) were considered the climatic locations. According to the Köppen–Geiger classification, the climate of Valença is humid subtropical (Cfa; Peel et al., 2007; Figure 1). The entrance of the natural microbial community into the stationary phase was considered the borderline of the method.

### 2.6 Validation results

After the initial analyses based on temperature, season, and agglomerative hierarchical clustering, a threshold was applied to the dendrogram. Consequently, metagenomic analyses, organic acid profiling, and culturing were conducted to assess the spoilage profile during the problematic season. Total DNA was extracted from both the sausages and the liquid present on the surface of the products inside the vacuum packages.

#### 2.6.1 High-throughput sequencing

Microbial community identification high-throughput sequencing was performed for sequencing the multiple microbial DNA molecules extracted from the sausages. The total microbial DNA of the sample groups (*N* = 3) was evaluated to determine the microbial agents responsible for sausage spoilage. The DNA of the samples was extracted using the Stool PSP Spin DNA kit (STRATEC Biomedical AG, Germany). V3–V4 primers were employed under the following conditions: the first polymerase chain reaction (PCR) primer with Illumina sequences based on the TruSeq structure adapter (Illumina, San Diego, CA) and the second PCR with indexing sequences. The first PCR reaction was carried out using Platinum Taq (Invitrogen, USA) at 95°C for 5 min, 95°C for 45 s, 55°C for 30 s, 72°C for 45 s (25 cycles), and a final extension of 72°C for 2 min. The second PCR was carried out at 95°C for 5 min, 95°C for 45 s, 66°C for 30 s, 72°C for 45 s (10 cycles), and a final extension of 72°C for 2 min. For comparison, the Illumina 16S protocol was employed (Illumina Technical Note 15044223 Rev. B). The final PCR reaction was cleaned up using AMPureXP beads (Beckman Coulter, Brea, CA), and the samples were pooled in the sequencing libraries for quantification. The pool amplicon estimations were performed using Picogreen dsDNA assays (Invitrogen, USA), and the pooled libraries were diluted for accurate qPCR quantification using the KAPA Library Quantification Kit for Illumina platforms (KAPA Biosystems, Woburn, MA). The libraries were sequenced in a MiSeq system using the standard Illumina primers provided in the kit. After sequencing, bioinformatics sequence demultiplexing, adaptor, and primer trimming were performed.

#### 2.6.2 Bioinformatic analysis

The bioinformatic analysis of the gut microbiota was performed using the DADA2 tool to analyze fastq sequences, aiming to recover single-nucleotide–resolved amplicon sequence variants (ASVs) from the amplicon data, following the methodology of Haas et al. (2022). This process resulted in a total of 146 ASVs. To remove the adapter sequences at the 5′ end, the trimLeft option was set to 17 for forward reads and 21 for reverse reads, following the methodology of Haas et al. (2022).

#### 2.6.3 Acetic and lactic acid compounds

Lactic, acetic, and citric acids were quantified using the same HPLC system but equipped with an Aminex HPX-87H column (ion exclusion, Bio-Rad) and a UV detector set to 210 nm. Standards for organic acids and sugars were obtained from Sigma-Aldrich (Steinheim, Germany) as described by Tlais et al. (2022).

### 2.7 Statistical analysis

The results related to the a_w_ and pH measurements were presented as mean ± standard error (SE) from triplicates. The durability of the samples was described using the microbial growth predictor MicroLab_ShelfLife, with natural microbial load, temperature, and a_w_ as fixed effects and random terms for the batches. The data were evaluated using analysis of variance (ANOVA), followed by Fisher's test (*p* < 0.05). Multiple factor analysis (MFA) was conducted to simultaneously evaluate the effects of the variables (initial microbial load, temperature fluctuation profile for each season, and a_w_) on the durability of the sausages. Similarities and discrepancies among the samples were grouped using agglomerative hierarchical clustering (AHC) and permutational analysis of variance (PERMANOVA), using the XLSTAT statistical and data analysis solution (Addinsoft, 2019), Boston, USA.

## 3 Results

The quality of meat products and sustainable production practices are pivotal topics in the food industry, especially concerning the microbiome resilience of sausages stored in vacuum systems with a high level of reduction in chemical preservatives. This study investigated the impact of biological profiles across different seasons on the microbiome stability of vacuum-packaged cooked sausages commercialized in Brazil. By analyzing 355 samples collected over four seasons, the research delved into parameters, such as pH, water activity, total lipids, and proteins, alongside microbial growth predictors and metagenomic analyses. From the *N* = 23 sample groups, *N* = 12 complied with the acceptance criterion considered in this study (#1, #3, #7, #8, #9, #12, #13, #14, #15, #16, #17, and #18).

The initial microbial load of a product is a critical determinant of its potential for spoilage (Table 1). The samples with initial microbial loads exceeding 6 log cfu/g were excluded from the study to ensure a manageable starting point for microbial growth. It is important to highlight that the Brazilian regulatory is considered to confirm our threshold (Table 1). High initial microbial loads can significantly shorten the shelf life of sausages by accelerating the spoilage process as microorganisms rapidly proliferate to spoilage levels. For instance, samples #2 (8.27 log cfu/g), #4 (8.88 log cfu/g), and #5 (8.81 log cfu/g) were eliminated due to their high initial microbial counts (Table 1).

**Table 1:** Initial microbial load and water activity (a_w_) measurements of the vacuum-packaged cooked sausages (mean ± standard error).

**Sample group**	**Initial microbial load (log cfu/g)**	**a_w_-value**	**pH-value**	**Criteria**
#1	3.76 ± 0.135	0.975 ± 0.002^a^	6.36 ± 0.040	Accepted
#2	8.27 ± 0.170	0.957 ± 0.005^b^	6.34 ± 0.020	Eliminated
#3	3.55 ± 0.381	0.962 ± 0.001^b^	6.46 ± 0.080	Accepted
#4	8.88 ± 0.140	0.972 ± 0.001^ab^	6.37 ± 0.120	Eliminated
#5	8.81 ± 0.132	0.968 ± 0.002^ab^	6.42 ± 0.040	Eliminated
#6	7.25 ± 0.210	0.935 ± 0.003^d^	6.42 ± 0.080	Eliminated
#7	5.25 ± 0.095	0.946 ± 0.002^cd^	6.39 ± 0.040	Accepted
#8	2.23 ± 0.216	0.94 ± 0.001^c^	6.34 ± 0.000	Accepted
#9	3.75 ± 0.118	0.943 ± 0.003^c^	6.52 ± 0.020	Accepted
#10	8.48 ± 0.181	0.973 ± 0.001^a^	6.44 ± 0.060	Eliminated
#11	7.75 ± 0.104	0.947 ± 0.003^cd^	6.29 ± 0.010	Eliminated
#12	5.75 ± 0.091	0.973 ± 0.003^a^	6.31 ± 0.070	Accepted
#13	4.76 ± 0.042	0.973 ± 0.003^a^	6.32 ± 0.150	Accepted
#14	5.99 ± 0.075	0.956 ± 0.003^b^	6.32 ± 0.090	Accepted
#15	4.01 ± 0.115	0.944 ± 0.001^c^	6.34 ± 0.040	Accepted
#16	5.01 ± 0.082	0.945 ± 0.003^c^	6.4 ± 0.050	Accepted
#17	2.35 ± 0.214	0.942 ± 0.002^c^	6.45 ± 0.040	Accepted
#18	5.62 ± 0.169	0.969 ± 0.001^ab^	6.5 ± 0.080	Accepted
#19	7.65 ± 0.062	0.953 ± 0.001^b^	6.42 ± 0.030	Eliminated
#20	9.14 ± 0.346	0.975 ± 0.002^a^	6.38 ± 0.010	Eliminated
#21	9.31 ± 0.306	0.979 ± 0.002^a^	6.39 ± 0.010	Eliminated
#22	7.23 ± 0.153	0.972 ± 0.004^a^	6.3 ± 0.060	Eliminated
#23	6.54 ± 0.064	0.97 ± 0.005^a^	6.42 ± 0.050	Eliminated

Different letters indicate significant differences using Fisher's (LSD) test (p < 0.05).

Therefore, groups #8 and #17 showed initial microbial loads below 3 log cfu/g. Groups #1, #3, #9, #13, and #15 presented intermediate microbial loads from 3 to 5 log cfu/g, while groups #7, #12, #14, #16, and #18 showed initial microbial loads from 5 to 6 log cfu/g.

Similarly, a_w_ is an important factor influencing microbial growth, with higher a_w_ values supporting faster microbial proliferation (Table 1). The accepted samples, such as #1 (a_w_ 0.975), #3 (a_w_ 0.962), and #7 (a_w_ 0.946), had a_w_ values in the lower range. In contrast, samples #10 (a_w_ 0.973), #21 (a_w_ 0.979), and #23 (a_w_ 0.970) were excluded due to their higher initial CFU/g values. Groups #7, #8, #9, #15, #16, and #17 had values below 0.95. Groups #3, #14, and #18 presented values ranging from 0.95 to 0.97, while the results above 0.97 were determined for groups #12 and #13 (Table 1).

The pH values did not differ among the groups, varying from 6.31 (#12) to 6.52 (#9; Table 1).

The present study determined the initial microbial load as a relevant factor related to sausage spoilage. Regardless of the a_w_ values, the groups with higher initial microbial loads (above 5 log cfu/g) showed lesser durability compared to those groups with the initial microbial loads below 5 log cfu/g. The combined factors of temperature fluctuation, initial microbial load, and a_w_ significantly influenced the durability of the vacuum-packaged cooked sausages, as shown in Figure 1. Temperature fluctuation is particularly important in the spoilage process. It is important to emphasize the necessity of consistent temperature control in preserving the quality and safety of perishable foods. In Brazil, sausages are often sold and displayed at varying temperatures in supermarkets, ranging from ideal cold storage to room temperature. This variation can lead to rapid microbial growth, affecting the product's shelf life and safety (Jansson et al., 2017; Teuteberg et al., 2021). Understanding these factors is important to enhance the durability and sustainability of meat products in the market.

**Figure 2:**
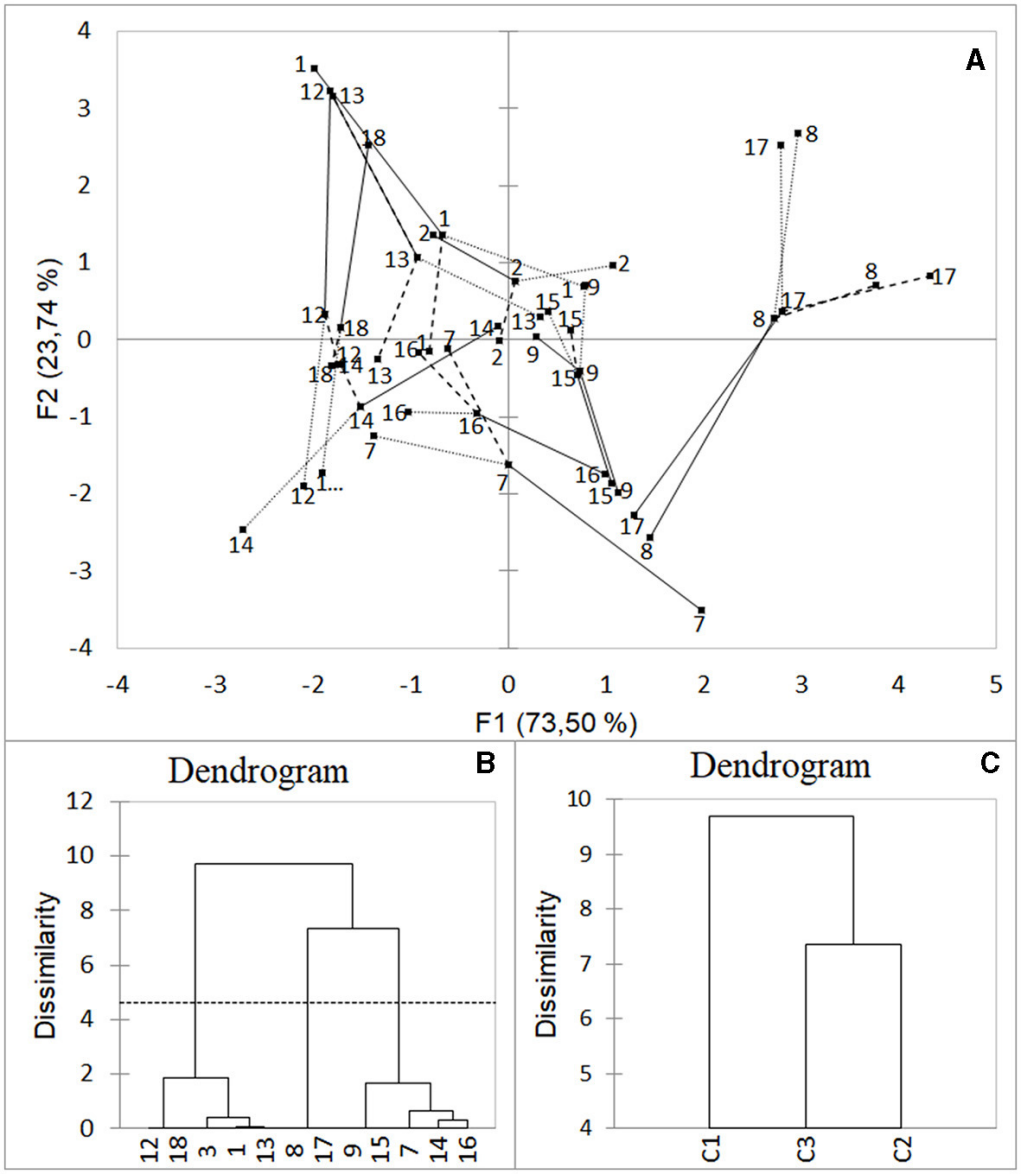
Influence of the temperature fluctuation for each season, initial microbial load, and a_w_ on the durability of the sausages. Multiple factor analysis **(A)** was conducted to randomly evaluate the effects of the variables (initial microbial load, a_w_, and daily temperature fluctuation per season). Similarities and discrepancies among the samples were grouped by agglomerative hierarchical clustering **(B, C)**, using the XLSTAT statistical and data analysis solution (Addinsoft, 2019), Boston, USA.

As indicated by the RV coefficients obtained from the MFA, the durability of the vacuum-packaged cooked sausages was mainly affected by the temperature (0.906), followed by the initial microbial load (0.755) and a_w_ (0.624; Figure 2A). Three clusters of similarity were observed, which were statistically (AHC) classified as C1 (Groups #1, #3, #12, #13, and #18), C2 (Groups #8 and #17), and C3 (Groups #7, #9, #14, #15, and #16; Figures 2B, C).

The present study demonstrated the relevance of cold chain management during the distribution of sausages, highlighting temperature and the season as a crucial parameter for extending the durability of vacuum-packaged cooked sausages. A PERMANOVA was carried out, which showed the season (*p* < 0.05). Cold storage may be considered the quickest and most effective way to improve the durability of vacuum-packaged cooked sausages.

Table 2 presents the total microbial count (log cfu/g) of the vacuum-packed cooked sausages across different samples and incubation conditions. The average microbial counts for each condition were as follows: initial condition (0) at 4.30 log cfu/g, 7°C for 4 days at 4.61 log cfu/g, 7°C for 8 days at 4.90 log cfu/g, 36°C for 4 days at 6.06 log cfu/g, and 36°C for 8 days at 6.79 log cfu/g. This analysis indicated that the condition of 36°C for 8 days resulted in the highest average microbial count, suggesting that microbial growth is most prolific under these conditions. Similarly, the condition of 36°C for 4 days also showed a high microbial count. On the other hand, the initial condition without incubation had the lowest microbial counts, indicating minimal microbial activity at baseline.

**Table 2:** Durability study of the vacuum-packaged cooked sausages in different seasons estimated by the predictive method.

**Incubation**	**#1**	**#3**	**#7**	**#8**	**#9**	**#12**	**#13**	**#14**	**#15**	**#16**	**#17**	**#18**
	**(#) Sample code—total microbial counting (log cfu/g)**											
0	3.76	3.55	5.25	2.23	3.75	5.75	4.76	5.99	4.01	5.01	2.35	5.62
7°C/4 days	3.99	3.75	5.24	2.45	3.71	6.23	5.25	6.56	4.1	5.23	2.55	5.99
7°C/8 days	4.01	3.91	5.77	2.65	3.89	6.89	5.58	6.54	4.09	5.55	2.57	6.13
36°C/4 days	6.76	5.98	6.21	3.67	5.32	7.01	6.52	7.52	5.01	6.34	3.67	7.43
36°C/8 days	6.89	6.45	7.32	4.75	6.67	7.55	7.15	7.83	7.23	7.15	4.63	7.46
	**Specific maximum growth rate (log cfu/g/day)**											
7°C	0.0444	0.0475	0.0313	0.0538	0.0037	0.1313	0.1125	0.1056	0.0163	0.0613	0.0388	0.0781
36°C	0.5706	0.4850	0.2494	0.3375	0.3788	0.2700	0.3694	0.3063	0.3263	0.3000	0.3075	0.3413
	**Ngrowth (log cfu/g/day)** ^*^											
*Summer*	0.4519	0.3863	0.2002	0.2735	0.2942	0.2387	0.3114	0.2610	0.2563	0.2461	0.2469	0.2819
*Autumn*	0.2644	0.2304	0.1224	0.1724	0.1605	0.1893	0.2199	0.1895	0.1459	0.1611	0.1511	0.1881
*Winter*	0.1737	0.1550	0.0848	0.1235	0.0959	0.1653	0.1756	0.1549	0.0924	0.1199	0.1048	0.1428
*Spring*	0.3113	0.2694	0.1419	0.1977	0.1939	0.2016	0.2428	0.2074	0.1735	0.1823	0.1751	0.2116
	**Ndeceleration (log cfu/g/day)** ^**^											
*Summer*	0.1295	0.1107	0.0574	0.0784	0.0843	0.0684	0.0892	0.0748	0.0735	0.0705	0.0707	0.0808
*Autumn*	0.1025	0.0893	0.0475	0.0668	0.0622	0.0734	0.0852	0.0734	0.0565	0.0624	0.0586	0.0729
*Winter*	0.0812	0.0724	0.0396	0.0577	0.0448	0.0773	0.0821	0.0724	0.0432	0.0560	0.0490	0.0667
*Spring*	0.1109	0.0960	0.0505	0.0704	0.0691	0.0718	0.0865	0.0739	0.0618	0.0649	0.0624	0.0754
	**Sausage's durability (days)** ^***^											
*Summer*	16	20	32	34	27	25	22	23	31	27	37	22
*Autumn*	26	32	45	50	44	27	27	25	47	37	56	28
*Winter*	38	45	61	67	70	29	32	28	70	46	78	34
*Spring*	24	28	41	45	38	27	26	24	41	33	49	26

^*^Daily microbial population growth (log cfu/g) in the L phase.

^**^Daily microbial population growth (log cfu/g) in the D phase.

^***^The entrance of the natural microbial community into the stationary phase was considered the borderline of the method.

In terms of individual sample quality, the samples from the 36°C for 8 days condition exhibited the highest microbial counts, with Sample #18 having the highest at 7.46 log cfu/g, followed by Sample #15 at 7.23 log cfu/g and Sample #14 at 7.15 log cfu/g. These high counts suggested that these samples were more prone to spoilage and had lower quality. Conversely, the samples from the initial condition (0) displayed the lowest microbial counts, with sample #8 having the lowest at 2.23 log cfu/g, followed by sample #17 at 2.35 log cfu/g and sample #9 at 3.75 log cfu/g, indicating better quality and longer shelf life. This analysis underscored the importance of incubation conditions in assessing the microbial quality of vacuum-packed cooked sausages. Different conditions significantly influence the microbial landscape and the overall product quality, highlighting the need for careful monitoring and control of incubation conditions for ensuring the safety and quality of these products.

Furthermore, Table 2 provides the durability of the vacuum-packed cooked sausages across different samples and seasons. The average durability for each season was calculated as follows: summer (26.33 days), autumn (37 days), winter (49.83 days), and spring (33.5 days). Winter showed the highest average durability, indicating that sausages stored during this season tend to last the longest under vacuum conditions. Autumn also displayed good durability, although not as high as winter. Spring and summer had lower average durability, with summer having the shortest average shelf life.

Comparing seasonal durability, winter (49.83 days) demonstrated significantly higher durability compared to summer (26.33 days), indicating that colder temperatures likely enhance the preservation of cooked sausages. Autumn (37 days) also showed better durability compared to spring (33.5 days), suggesting that summer conditions, as compared to spring conditions, are more favorable for sausage preservation. The individual sample analysis revealed that the highest durability was observed in winter, with sample #17 lasting 78 days, followed by sample #9 lasting 70 days. Conversely, the lowest durability was observed in summer, with sample #1 lasting only 16 days and sample #18 lasting 22 days. These data underscored the importance of temperature control and seasonality in preserving sausage quality, highlighting the need for tailored storage strategies for ensuring optimal product quality and shelf life throughout the year.

The cost and environmental impact associated with food waste due to the early deterioration of sausages does not justify the energy savings achieved in the markets.

Many efforts have been reported in the literature to improve the durability of vacuum-packaged cooked sausages, which include the selection of raw material, microbiological process control, and post-package treatment, among others. In this study, it is clear that even when all evaluated factors are considered concomitantly, failures regarding sausage preservation may occur if the cold chain management is poor.

Corroborating with the results shown in Figure 2, temperature is a factor of significant impact on microbial growth in vacuum-packaged cooked sausages. It may be demonstrated by the results presented in Table 3 regarding the resilience study.

**Table 3:** Durability study of the vacuum-packaged cooked sausages during cold storage (7°C) estimated by the predictive method.

**Incubation**	**#1**	**#3**	**#7**	**#8**	**#9**	**#12**	**#13**	**#14**	**#15**	**#16**	**#17**	**#18**
	**(#) Samples code—total microbial counting (log cfu/g)**											
0	3.76	3.55	5.25	2.23	3.75	5.75	4.76	5.99	4.01	5.01	2.35	5.62
7°C/4 days	3.99	3.75	5.24	2.45	3.71	6.23	5.25	6.56	4.1	5.23	2.55	5.99
7°C/8 days	4.01	3.91	5.77	2.65	3.89	6.89	5.58	6.54	4.09	5.55	2.57	6.13
36°C/4 days	6.76	5.98	6.21	3.67	5.32	7.01	6.52	7.52	5.01	6.34	3.67	7.43
36°C/8 days	6.89	6.45	7.32	4.75	6.67	7.55	7.15	7.83	7.23	7.15	4.63	7.46
	**Specific maximum growth rate (log cfu/g/day)**											
7°C	0.0444	0.0475	0.0313	0.0538	0.0037	0.1313	0.1125	0.1056	0.0163	0.0613	0.0388	0.0781
36°C	0.5706	0.485	0.2494	0.3375	0.3788	0.2700	0.3694	0.3063	0.3263	0.3000	0.3075	0.3413
	**Ngrowth (log cfu/g/day)** ^*^											
	0.0444	0.0475	0.0313	0.0538	0.0037	0.1313	0.1125	0.1056	0.0163	0.0613	0.0388	0.0781
	**Ndeceleration (log cfu/g/day)** ^**^											
	0.0293	0.0314	0.0207	0.0355	0.0025	0.0867	0.0744	0.0698	0.0107	0.0405	0.0256	0.0516
	**Sausagess' durability (days)** ^***^											
	138	133	147	142	>180	32	46	37	>180	79	>180	54

^*^Daily microbial population growth (log cfu/g) in the L phase.

^**^Daily microbial population growth (log cfu/g) in the D phase.

^***^The entrance of the natural microbial community into the stationary phase was considered the borderline of the method.

Table 3 provides the durability (in days) of the vacuum-packed cooked sausages stored at 7°C across different samples. The average durability at this temperature was ~108.33 days. Samples #9, #15, and #17 exhibited the highest durability, each lasting over 180 days, indicating exceptional quality and resistance to spoilage. Conversely, sample #12 had the lowest durability at 32 days, followed by sample #14 at 37 days and sample #13 at 46 days, suggesting that these samples were more prone to spoilage. This analysis highlighted that while storing sausages at 7°C was generally effective in extending the shelf life, with an average durability of over 100 days, there was significant variability among the individual samples, necessitating careful monitoring to ensure consistent quality.

Samples #8 and #17, which presented the initial microbial loads and a_w_ below 3 log cfu/g and 0.95, respectively, showed higher durability than samples #14 and #16, which showed the initial microbial loads between 5 and 6 log cfu/g and a_w_ above 0.97. Among the samples with the initial microbial loads between 5 and 6 log cfu/g (#7, #12, #14, #16, and #18), samples #7 and #16 (a_w_ below 0.95) presented higher durability than samples #12, #14, and #18 (a_w_ above 0.95). Similar trends were observed in the groups with the initial microbial loads between 3 and 5 log cfu/g (Table 2).

The samples with an a_w_ value above 0.95 showed less durability than the groups with an a_w_ value below 0.95, even with close initial microbial loads. This could be observed in sample #7 (initial microbial load of *ca*. 5.25 log cfu/g; a_w_ = 0.946 ± 0.002) and sample #18 (initial microbial load of *ca*. 5.62 log cfu/g; a_w_ = 0.969 ± 0.001). Considering summer as the most critical period, the durability presented by sample #7 was 10 days more than that of sample #18. As the temperature was entered in the model as a fixed variable and the initial microbial loads were close, the difference in the resilience may be attributed to the a_w_ values.

The effect of temperature on the spoilage of vacuum-packaged cooked sausage may be potentialized by sausage recipes that confer to the product's high initial microbial load and a_w_. The natural microbiota of vacuum-packaged cooked sausages is predominantly dominated by mesophilic groups, and microbial growth easily occurs at a_w_ above 0.94.

Samples #12, #13, #14, and #18 were little affected when the temperature was reduced to 7°C. Therefore, it was assumed that there was a presence of psychrotrophic microorganisms at high loads in these groups. Considering that psychrotrophic microorganisms are also part of the natural microbiota of meat products, and recognizing the challenges associated with managing the cold chain, it is advisable for sausage makers to improve their recipes to reduce the initial microbial load and a_w_ of products (Food Preservation by Reducing Water Activity, 2016). In microbial growth curves simulated by MicroLab_ShelfLife, the combination of a_w_ below 0.95 and cold chain management temperature fitted the microbial growth curve to a linear profile in the L phase, as shown in Supplementary Figures 1–4 (#7, #8, #9, #15, and #17), postponing the entrance of the microbiota into the stationary phase and hindering microbial growth. Thus, producers should consider recipes with a_w_ below 0.95 and an initial microbial load of *ca*. 3.0 log cfu/g.

**Figure 3:**
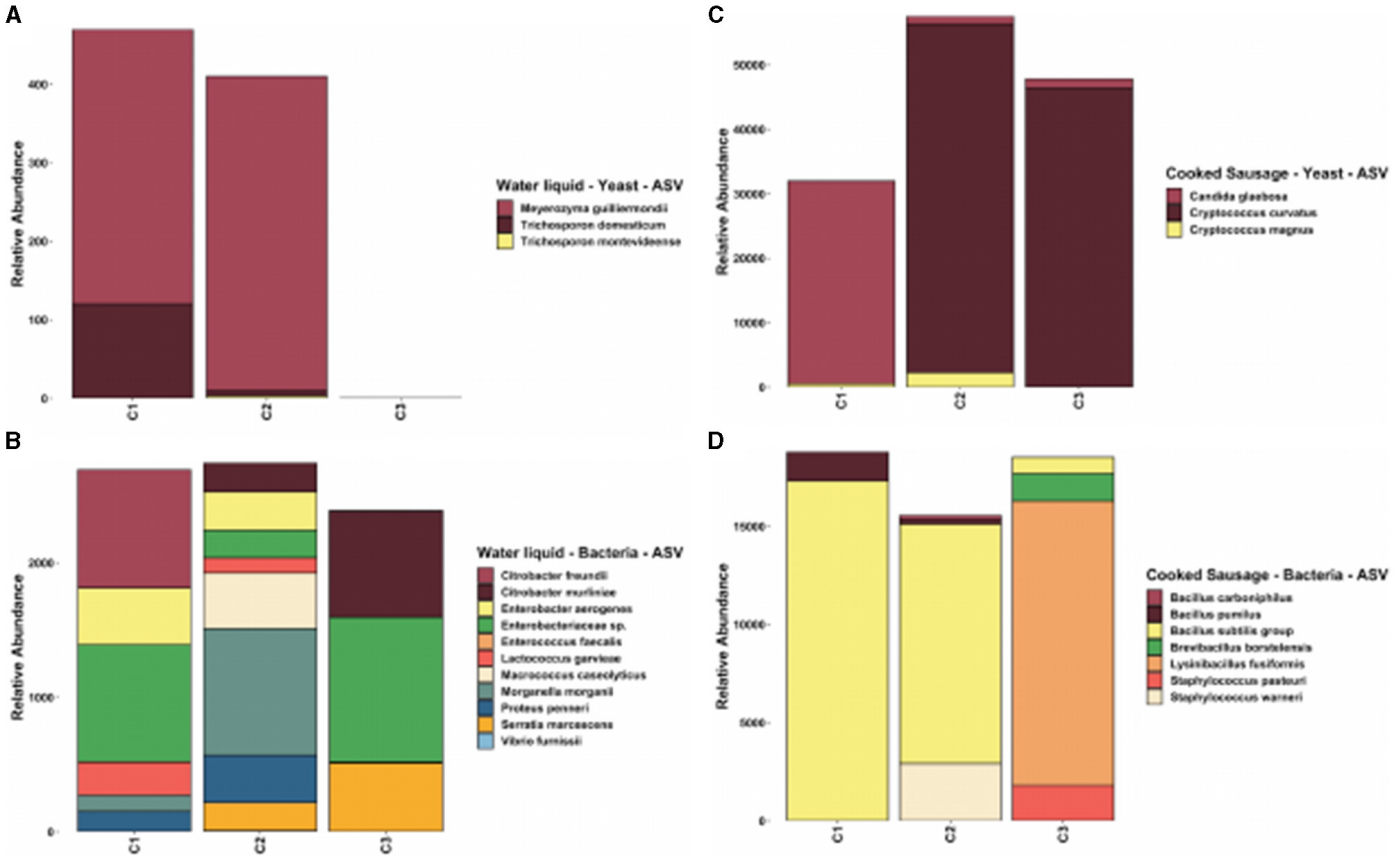
Relative abundance **(A)** yeast diversity in the water liquid, **(B)** bacterial diversity in the water liquid, **(C)** yeast diversity in the cooked sausages, and **(D)** bacterial diversity in the cooked sausages.

In the results of this experimental design, three clusters were observed (Figure 2C). Consequently, a metagenomic analysis was conducted to identify the microbiome profile indicative of spoilage in the product. The metagenomic analysis revealed low diversity in the sausage samples, as expected due to the cooking process, rendering traditional statistical models inapplicable (Figure 3 and Supplementary Table 1). Cluster C2 exhibited higher syneresis activities based on a_w_, which were evident in the yeast density, particularly the *Trichosporon* genus (95%) found in the covering liquid. This microorganism is commonly present in the gastrointestinal tract, respiratory tract, and skin (Colombo et al., 2011).

Figure 3 was generated without normalization to avoid potential misunderstandings as the diversity confirmed that the cluster analysis was performed correctly and that there were differences among the three selected clusters.

In the water liquid, *Trichosporon domesticum* dominated sample C1 with 25.5% of the yeast population but was absent in sample C3. Meanwhile, *Meyerozyma guilliermondii* was prevalent in both samples C1 (74.5%) and C2 (97.6%), indicating its strong role in spoilage. *Trichosporon montevideense* and *M. guilliermondii* were notably absent in sample C3, where the latter solely constituted 100% of the yeast population, reflecting specific environmental conditions favoring its growth in isolation.

In sample C1, *Citrobacter freundii* and *Enterobacteriaceae* dominated the bacterial population, contributing significantly to potential spoilage. Sample C2 featured *Morganella morganii* as the most abundant species at 34.4%, highlighting its impact on spoilage dynamics alongside *Proteus penneri* and *Macrococcus caseolyticus*. In sample C3, *Enterobacteriaceae* and *Citrobacter murliniae* were prevalent, with *Serratia marcescens* contributing to the complex microbial ecosystem affecting product stability.

In the cooked sausages, *Candida glaebosa* dominated sample C1 with 98.3%, while *Cryptococcus magnus* and *Cryptococcus curvatus* were present in samples C2 and C3, highlighting their adaptability to different conditions. The presence of *C. curvatus* in samples C2 and C3 suggested environmental conditions conducive to its growth, contributing significantly to spoilage. The variation in the yeast species across samples emphasizes the need for targeted microbial control strategies in sausage preservation.

The bacterial landscape in the cooked sausages showed a strong presence of the *Bacillus subtilis* group, dominating samples C1 and C2 at 92.0 and 78.5%, respectively. Sample C3 was primarily composed of *Lysinibacillus fusiformis*, with other bacteria such as *Staphylococcus pasteuri* and *Brevibacillus borstelensis* playing secondary roles. This distribution highlights the complex microbial dynamics influencing spoilage and underscores the need for improved microbial management for enhancing the product's shelf life and safety.

The microbial composition between the product and label, and within the cooked sausages, revealed significant variations that can impact spoilage and quality. In the water liquid, the dominance of *Trichosporon domesticum* in sample C1 and *M. guilliermondii* in samples C1 and C2 indicated yeast proliferation as a spoilage risk. Bacterial analysis showed that *Citrobacter freundii* and *Enterobacteriaceae* were prevalent in the water liquid, while *Bacillus subtilis* dominated the sausages, particularly in samples C1 and C2, highlighting their roles in spoilage. The presence of *C. curvatus* in samples C2 and C3 sausages suggests a need for targeted interventions to control microbial growth and extend product shelf life.

**Figure 4:**
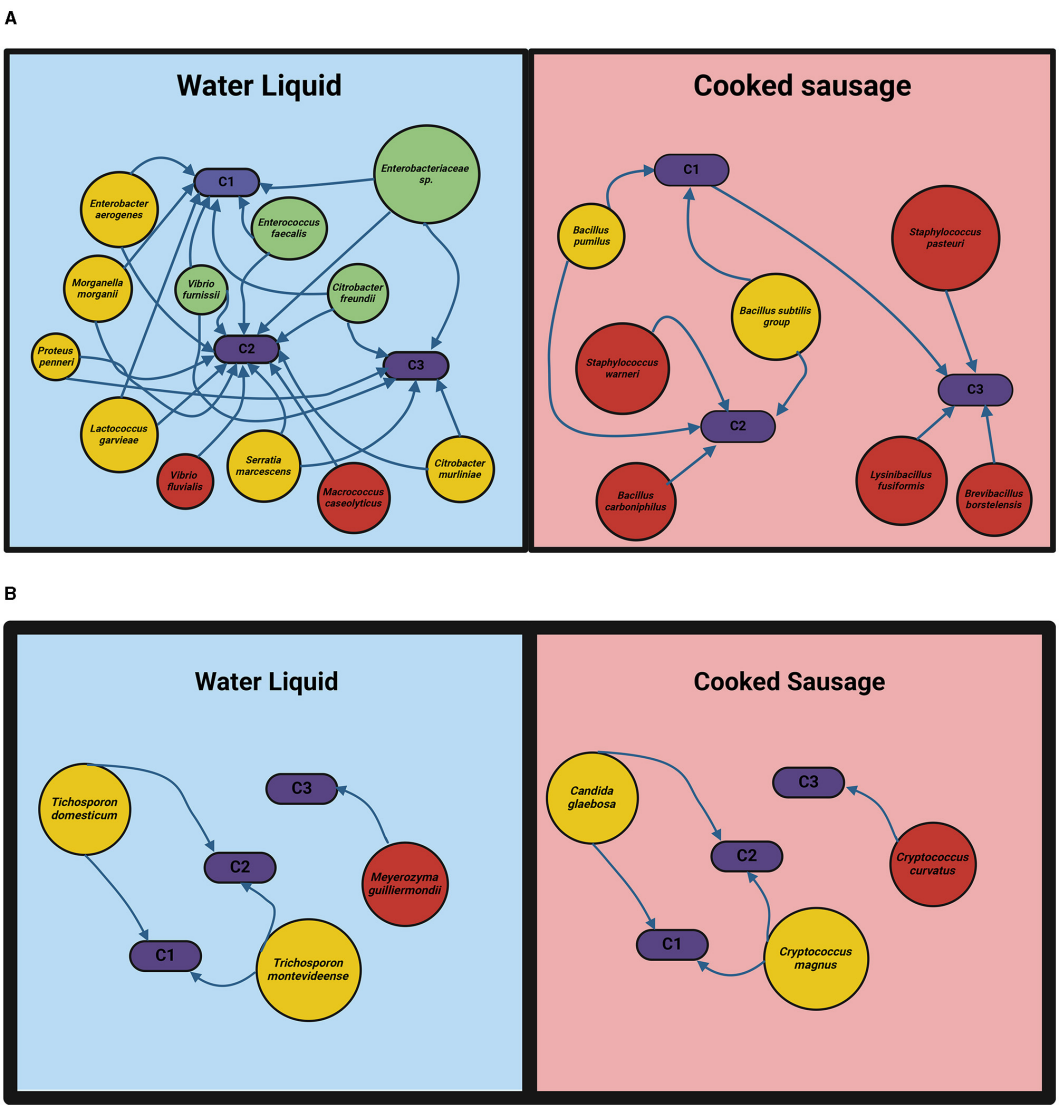
The figure illustrates the microbial interaction networks for bacteria **(A)** and yeasts **(B)** in the water liquid and cooked sausage environments. In the bacterial network, red circles represent the species with only one interaction, yellow circles represent the species with two interactions, and green circles denote the species with three interactions.

The network (Figure 4A) on the left shows the various bacteria identified in the water liquid, with *Enterobacteriaceae, Enterococcus faecalis, Vibrio furnissii*, and *Citrobacter freundii* being the groups with the most interactions with C1, C2, and C3. In the cooked sausage network on the right, the *B. subtilis* group and *S. pasteuri* are highlighted as significant players present in the groups C1 and C2. This can be associated with the sustainable ingredients added as *Bacillus* is commonly found in the environment and frequently used as a biocontrol agent (Chen et al., 2020).

Figure 4B presents the yeast interaction networks in the water liquid and cooked sausage. In the water liquid, *T. domesticum* and *T. montevideense* were the key yeasts with the most interactions with C1 and C2, while *Meyerozyma guilliermondii* also played a significant role with fewer interactions with C3. In the cooked sausage, *Candida glaebosa* and *C. magnus* were prominent with multiple interactions with C1 and C2, with *C. curvatus* presentwith fewer interactions with C3.

The microbial composition in each sample was influenced by the a_w_ levels, which affected the growth and dominance of specific species. C1, with a higher a_w_ value of 0.9704, supported the proliferation of *M. guilliermondii* and *B. subtilis*, both known for thriving in high-moisture environments. C2, with a lower a_w_ value of 0.941, showed dominance by *Morganella morganii* and *C. curvatus*, reflecting a microbial community adapted to less moisture.

C3, with an a_w_ of 0.9468, facilitated the presence of *Enterobacteriaceae* and *Lysinibacillus fusiformis*, indicating a balanced environment that supports diverse microbial populations and spoilage dynamics.

## 4 Discussion

The shelf life of a food product is the period within which a food maintains its safety and quality under reasonably foreseeable conditions of distribution, storage, and use (Guidande Note No. 18, 2019). Microbial growth is the main cause of meat product spoilage during distribution and disposal for sale in markets (Cauchie et al., 2020).

Numerous aspects, such as the type and quality of raw materials used in sausage recipes, the environment, the handlers and utensils, and the natural meat microorganisms, may impact the initial microbial load of sausages (Korkeala and Björkroth, 1997). Once no post-packaging treatment is applied, cooking is the only treatment that may significantly impact the initial microbial load. Although external hurdle products are generally added as preservatives, they only play a bacteriostatic role (Rogovski et al., 2021).

Among the factors studied in this research, sausage spoilage was less impacted by a_w_. However, it may act as a trigger for the spoilage process of vacuum-packaged cooked sausages. Conditions of a_w_ above 0.95, post-package handling, and negative pressure, which is observed in vacuum packaging, may induce product syneresis after packaging, resulting in the accumulation of water, nutrients, and microorganisms inside the package. Preservatives are usually added to the mass with other ingredients, which results in their partial migration to the liquid phase (de Lima et al., 2022). This factor can be decisive in accelerating microbial growth when the product is exposed to an abusive temperature. Thus, the effect of temperature on microbial growth may be minimized by preventing syneresis in the product. Specifically, summer is the critical period resulting in product spoilage in tropical climate countries, where the average temperature during the season may be close to the optimum temperature for the growth of mesophilic microorganisms (Teuteberg et al., 2021). Ranucci et al. (2019) showed that cold storage of vacuum-packaged cooked sausages keeps the total mesophilic bacteria count below 7.0 log cfu/g for a duration of 60 days. Therefore, the entrance of the natural microbial community into the stationary phase is considered the borderline of the microbial growth predictor named MicroLab_ShelfLife. The method can provide an estimation of the natural microbial growth in a plotted predictive curve (Guerra et al., 2023). However, the activity of the microbiota is out of the sensitivity of the method. Therefore, the real impact of the initial microbial load may be more severe as microbial activity may not be associated with population growth (Colarusso et al., 2021). Godoy et al. (2022) demonstrated the effect of cold chain management on the chicken sausage spoilage process, revealing enhancements of 75 days in the durability of sausages. Therefore, markets are the most critical point throughout the logistic distribution of the products where the cold chain is poorly managed.

In addition, a study on Salame Piemonte, an Italian PGI fermented sausage, revealed that strain-level biodiversity significantly influences the quality of the final product. The authors identified *Pediococcus pentosaceus, Latilactobacillus curvatus*, and *Latilactobacillus sakei* as the dominant lactic acid bacteria, with distinct strains of *L. sakei* affecting the fermentation process and sensory characteristics through varied metabolic pathways. The research highlighted the importance of strain-level diversity in determining the sensory attributes of fermented foods (Franciosa et al., 2021).

In another study, the addition of 0.05% gallic acid (GA) to reduced-nitrite Chinese fermented sausages was shown to effectively decrease biogenic amines (BAs) and N-nitrosodimethylamine levels. The researchers found that GA promoted *Lactococcus* growth while reducing spoilage bacteria, leading to an 89.86% reduction in N-nitrosodimethylamine by regulating amino acids and trimethylamine pathways. The study underscored GA's potential as a plant-based alternative to control BAs and nitrosamines in meat products with reduced nitrite usage (Zhou et al., 2024).

It is important to highlight that cooked meat presents different nutritional compositions and microbial diversity. Hence, it was challenging to compare our results with those of other studies. Therefore, our discussion included comparisons with similar products that were not cooked.

Research on traditional fuet fermented sausages and inoculated fermented sausages (IFSs) showed that IFSs, deficient in diverse *Staphylococci*, failed to reproduce the volatilome profile of traditional fermented sausages (FSs). The presence of *Staphylococcus* species, such as *S. carnosus* and *S. xylosus*, alongside *Pediococcus*, was crucial for flavor compound production and nutrient levels. In addition, the study highlighted that *P. acidilactici* and *S. carnosus* in IFSs increased pyridoxal and indolelactic acid, linked to vitamin B6 and tryptophan pathways, emphasizing the importance of microbial diversity for authentic fermentation (Yang et al., 2022).

An investigation into traditional Chinese fermented meat products identified *Staphylococcus, Tetragenococcus*, and *Latilactobacillus* as the most abundant genera. The authors also pinpointed key antibiotic resistance genes (ARGs), *lnuA* and *abeM*, with *Staphylococcus, Acinetobacter*, and *Pseudomonas* serving as significant reservoirs of resistance (Teuteberg et al., 2021).

A study on the beef processing chain revealed dynamic changes in microbial communities, identifying key species, such as *Brochothrix thermosphacta, Carnobacterium maltaromaticum, Pseudomonas fragi, Psychrobacter cryohalolentis*, and *Psychrobacter immobilis*. The carcass samples and slaughterhouse surfaces showed high abundances of antibiotic resistance genes (ARGs) from various antibiotic classes, suggesting the potential for ARG transmission to human pathogens (Sequino et al., 2024).

Finally, research using 16S rRNA amplicon sequencing revealed that *Acinetobacter* and *Pseudomonas* were dominant on meat-contacted surfaces, with chicken-contacted trays showing the highest species richness. Key bacterial markers, including potential human pathogens such as *Shewanella* in chicken, *Staphylococcus* in beef, and *Klebsiella* in pork, were identified. The study highlighted the necessity for stringent hygiene practices and regular cleaning of food preparation areas to prevent cross-contamination and improve food safety (Sharafi et al., 2024).

These findings emphasize the profound impact of metagenomic approaches in unveiling the complex dynamics of microbial communities in various food products. By identifying previously neglected microorganisms and their roles in food safety and quality, metagenomics provides a deeper understanding of microbial ecosystems.

Temperature and a_w_ are critical factors affecting the quality, safety, and shelf life of vacuum-packaged cooked sausages. Elevated temperatures create optimal conditions for the growth of mesophilic bacteria, such as *Bacillus* and *Clostridium* species, which are common spoilage organisms in meat products (Li et al., 2020).

These microorganisms can proliferate rapidly under favorable conditions, leading to spoilage and potential food safety risks due to toxin production (Li et al., 2024). Although low temperatures can slow microbial growth, psychrotrophic bacteria, such as *Pseudomonas* sp., can thrive under refrigeration, causing spoilage even at reduced temperatures (Zhang et al., 2019).

However, in our study, the cooked sausages did not show the presence of this species. Therefore, maintaining appropriate storage temperatures is essential for controlling the microbial load and ensuring the quality of this type of meat product.

The a_w_ level influences microbial growth by affecting the availability of free water necessary for microbial metabolism. Meat products with high a_w_ levels (above 0.95) provide an ideal environment for the growth of bacteria and yeasts, accelerating spoilage processes (Taormina and Sofos, 2014). The present study highlighted that clusters with higher a_w_ values, such as those dominated by *M. guilliermondii* and *B. subtilis*, are more prone to rapid spoilage, emphasizing the need for a_w_ reduction strategies in meat preservation. Techniques such as curing and drying reduce a_w_, inhibiting the growth of spoilage microorganisms and extending the shelf life of the product (Taormina and Sofos, 2014).

Among the factors included in the present study, temperature should be fixed without high energy optimization regarding storage management.

Several instances are involved in product distribution, including product retention in the industry and markets, transport, and disposal for sale (Hundy et al., 2016). The use of air conditioners in markets usually exerts a slight temperature control during the product display for sale. However, the retention of products in warehouses remains to be controlled. It is also common to observe failures in temperature management during transport and in logistic centers used for product distribution (Godoy et al., 2022). Otherwise, cold chain management is usually not considered in the logistic distribution and retention of vacuum-packaged cooked sausages.

However, proper cold chain management may not be sufficient to extend the durability of vacuum-packaged cooked sausages when the microbiota predominantly consists of psychrotrophic microorganisms (Vargas et al., 2022).

It is important to highlight that a study found that spray-dried raspberry powder enhances the oxidative and microbial stability of vacuum-packed ground beef, improving the shelf life of the product by reducing lipid oxidation and improving color stability. However, further studies are needed to evaluate the substitution of other natural preservatives to improve shelf life and assess the effects of temperature, a_w_, and seasonal variations on meat quality (Aksu et al., 2023).

## 5 Conclusion

This study extensively analyzed the quality and microbial safety of vacuum-packaged cooked sausages stored under varying conditions over a 1-year period. We identified key factors that influence the resilience and spoilage dynamics of these products by employing a combination of omics techniques, microbiological assessments, and physicochemical analyses. Our findings demonstrated that storage temperature, initial microbial load, and a_w_ are crucial determinants of sausage durability, with the highest microbial counts recorded at 36°C for 8 days and the lowest in the initial condition. The seasonal analysis revealed that the sausages had the highest average durability in winter and the lowest in summer. The identification of yeast genera, such as *Trichosporon* and *Candida*, along with *Bacillus* species as dominant spoilage microorganisms, highlighted their significant role in spoilage processes.

The control of temperature and a_w_ is important for preventing spoilage and ensuring the quality of vacuum-packaged cooked sausages. The implementation of effective a_w_ reduction strategies, along with maintaining optimal storage conditions, can help mitigate the growth of spoilage microorganisms, thereby extending the shelf life of these products.

The results emphasize the importance of optimizing storage conditions, particularly maintaining cold temperatures, to ensure the microbial stability and safety of vacuum-packaged cooked sausages. Supermarkets and other retailers must adhere to these recommended storage procedures to prolong shelf life and prevent spoilage. Omics approaches, including high-throughput sequencing (ITS and 16S amplicon), have proven to be invaluable in uncovering the complex microbial interactions that impact product quality. These insights are critical for developing effective strategies to mitigate spoilage and enhance the shelf life of meat products within a sustainable supply chain.

## Data Availability

Data sets are publicly available at NCBI under the accession number (PRJNA1155639).

## References

[B1] Addinsoft XLSTAT. (2019). Data Analysis and Statistics With Microsoft Excel. Paris.

[B2] Aksu M. I. Turan E. Gülbandilar A. Tamtürk F. (2023). Utilization of spray-dried raspberry powder as a natural additive to improve oxidative stability, microbial quality and overcome the perception of discoloration in vacuum-packed ground beef during chilled storage. Meat Sci. 197:109072. 10.1016/j.meatsci.2022.109072 36516591

[B3] Barcenilla C. Álvarez-Ordóñez A. López M. Alvseike O. Prieto M. (2022). Microbiological safety and shelf-life of low-salt meat products-a review. Foods 11:52331. 10.3390/foods11152331 35954097 PMC9367943

[B4] Caccialanza A. Cerrato D. Galli D. (2023). Sustainability practices and challenges in the meat supply chain: a systematic literature review. Br. Food J. 125, 4470–4497. 10.1108/BFJ-10-2022-0866

[B5] Cauchie E. Delhalle L. Taminiau B. Tahiri A. Korsak N. Burteau S.. (2020). Assessment of spoilage bacterial communities in food wrap and modified atmospheres-packed minced pork meat samples by 16S rDNA metagenetic analysis. Front. Microbiol. 10:3074. 10.3389/fmicb.2019.03074 32038536 PMC6985204

[B6] Chen K. Tian Z. He H. Long C. Jiang F. (2020). Bacillus species as potential biocontrol agents against citrus diseases. Biol. Control 151:104419. 10.1016/j.biocontrol.2020.104419

[B7] Colarusso A. V. Goodchild-Michelman I. Rayle M. Zomorrodi A. R. (2021). Computational modeling of metabolism in microbial communities on a genome-scale. Curr. Opin. Syst. Biol. 26, 46–57. 10.1016/j.coisb.2021.04.001

[B8] Colombo A. L. Padovan A. C. B. Chaves G. M. (2011). Current knowledge of *Trichosporon* spp. and Trichosporonosis. Clin. Microbiol. Rev. 24, 682–700. 10.1128/CMR.00003-11 21976604 PMC3194827

[B9] de Lima A. L. Guerra C. A. Costa L. M. de Oliveira V. S. Lemos Junior W. J. Luchese R. H.. (2022). A natural technology for vacuum-packaged cooked sausage preservation with potentially postbiotic-containing preservative. Fermentation 8:30106. 10.3390/fermentation8030106

[B10] FAO (2021). Meat Market Review, Overview of Global Meat Market Developments in 2020 (Rome: Food and Agriculture Organization of the United Nations), 15.

[B11] Ferrocino I. Bellio A. Giordano M. Macori G. Romano A. Rantsiou K.. (2018). Shotgun metagenomics and volatilome profile of the microbiota of fermented sausages. Appl. Environ. Microbiol. 84:17. 10.1128/AEM.02120-17 29196291 PMC5772244

[B12] Food Preservation by Reducing Water Activity (2016). Food Microbiology: Principles into Practice, 44–58.

[B13] Franciosa I. Ferrocino I. Giordano M. Mounier J. Rantsiou K. Cocolin L. (2021). Specific metagenomic asset drives the spontaneous fermentation of Italian sausages. Food Res. Int. 144:110379. 10.1016/j.foodres.2021.110379 34053518

[B14] Giraffa G. Neviani E. (2001). DNA-based, culture-independent strategies for evaluating microbial communities in food-associated ecosystems. Int. J. Food Microbiol. 67, 19–34. 10.1016/S0168-1605(01)00445-7 11482566

[B15] Godoy C. A. L. Costa L. M. Guerra C. A. de Oliveira V. S. de Paula B. P. Lemos Junior W. J.. (2022). Potentially postbiotic-containing preservative to extend the use-by date of raw chicken sausages and semifinished chicken products. Sustainability 14:52646. 10.3390/su14052646

[B16] Guerra C. A. Costa L. M. de Oliveira V. S. de Paula B. P. Junior W. J. F. L. Luchese R. H.. (2023). Correlation between natural microbial load and formation of ropy slime affecting the superficial color of vacuum-packaged cooked sausage. Meat Sci. 201:109197. 10.1016/j.meatsci.2023.109197 37116267

[B17] Guidande Note No. 18 (2019). Validation of Product Shelf-Life., Revision 4. Dublin: Food Safety Authority of Ireland.

[B18] Haas E. A. Saad M. J. Santos A. Vitulo N. Lemos J.r, W. J. Martins A. M.. (2022). A red wine intervention does not modify plasma trimethylamine N-oxide but is associated with broad shifts in the plasma metabolome and gut microbiota composition. Am. J. Clin. Nutr. 116, 1515–1529. 10.1093/ajcn/nqac286 36205549 PMC9761755

[B19] Hundy G. F. Trott A. R. Welch T. C. (2016). “Chapter 17—the cold chain—transport, storage, retail,” eds. G. F. Hundy, A. R. Trott, and T. C. B. T.-R. *Welch Air Conditioning and Heat Pumps, 5th Edn* (Oxford: Butterworth-Heinemann), 273–287.

[B20] Ishangulyyev R. Kim S. Lee S. H. (2019). Understanding food loss and waste-why are we losing and wasting food? Foods 8:80297. 10.3390/foods8080297 31362396 PMC6723314

[B21] Iulietto M. F. Sechi P. Borgogni E. Cenci-Goga B. T. (2015). Meat spoilage: a critical review of a neglected alteration due to ropy slime producing bacteria. Ital. J. Anim. Sci. 14:4011. 10.4081/ijas.2015.4011

[B22] James S. J. James C. (2014). “Cooking of meat. Heat processing methods,” eds. M. Dikeman and C. B. T.-E. of M. S. Second E. Devine (Oxford: Academic Press), 385–390.

[B23] Jansson J. Nilsson J. Modig F. Hed Vall G. (2017). Commitment to sustainability in small and medium-sized enterprises: the influence of strategic orientations and management values. Bus. Strategy Environ. 26, 69–83. 10.1002/bse.1901

[B24] Kharola S. Ram M. Kumar Mangla S. Goyal N. Nautiyal O. P. Pant D.. (2022). Exploring the green waste management problem in food supply chains: a circular economy context. J. Clean. Prod. 351:131355. 10.1016/j.jclepro.2022.131355

[B25] Knipe C. L. (2014). “Sausages, types of cooked,” eds. M. Dikeman and C. B. T.-E. of M. S. Second E. Devine (Oxford: Academic Press), 241–247.

[B26] Korkeala H. J. Björkroth K. J. (1997). Microbiological spoilage and contamination of vacuum-packaged cooked sausages. J. Food Protect. 60, 724–731. 10.4315/0362-028X-60.6.724 31195570

[B27] Kumar M. Raut R. D. Jagtap S. Choubey V. K. (2023). Circular economy adoption challenges in the food supply chain for sustainable development. Bus. Strategy Environ. 32, 1334–1356. 10.1002/bse.319137406496

[B28] Li H. Sun X. Liao X. Gänzle M. (2020). Control of pathogenic and spoilage bacteria in meat and meat products by high pressure: challenges and future perspectives. Comp. Rev. Food Sci. Food Safe 19, 3476–3500. 10.1111/1541-4337.12617 33337070

[B29] Li M. Liang D. Liu S. Guo S. Li M. Zhu Y.. (2024). Analysis of spore prevalence and sporulation potential in prepackaged meat products. LWT 201:116150. 10.1016/j.lwt.2024.116150

[B30] Li X. Zhang R. Hassan M. M. Cheng Z. Mills J. Hou C.. (2022). Active packaging for the extended shelf-life of meat: perspectives from consumption habits, market requirements and packaging practices in China and New Zealand. Foods 11:182903. 10.3390/foods11182903 36141031 PMC9506090

[B31] Lonergan S. M. Topel D. G. Marple D. N. (2019). “Chapter 14—sausage processing and production,” eds. S. M. Lonergan, D. G. Topel, and D. N. B. T.-T. S. of A. G. and M. T. Second E. Marple (Oxford: Academic Press). 229–253.

[B32] Majou D. Christieans S. (2018). Mechanisms of the bactericidal effects of nitrate and nitrite in cured meats. Meat Sci. 145, 273–284. 10.1016/j.meatsci.2018.06.013 30005374

[B33] Morales P. Aguirre J. Troncoso M. Figueroa G. (2020). Comparison of *in vitro* and *in situ* antagonism assays as tools for the selection of bio-preservative lactic acid bacteria (LAB) in poultry meat. LWT 118:108846. 10.1016/j.lwt.2019.108846

[B34] Peel M. C. Finlayson B. L. McMahon T. A. (2007). Updated world map of the Köppen-Geiger climate classification. Hydrol. Earth Syst. Sci. 11, 1633–1644. 10.5194/hess-11-1633-2007

[B35] Ranucci D. Roila R. Andoni E. Braconi P. Branciari R. (2019). *Punica granatum* and *Citrus* spp. extract mix affects spoilage microorganisms growth rate in vacuum-packaged cooked sausages made from pork meat, emmer wheat (*Triticum dicoccum* Schübler), Almond (*Prunus dulcis* Mill.), and Hazelnut (*Corylus avellana* L.). Foods 8:120664. 10.3390/foods8120664 31835622 PMC6963912

[B36] Rogovski P. Cadamuro R. D. da Silva R. de Souza E. B. Bonatto C. Viancelli A.. (2021). Uses of bacteriophages as bacterial control tools and environmental safety indicators. Front. Microbiol. 12:793135. 10.3389/fmicb.2021.793135 34917066 PMC8670004

[B37] Sequino G. Cobo-Diaz J. F. Valentino V. Tassou C. Volpe S. Torrieri E.. (2024). Microbiome mapping in beef processing reveals safety-relevant variations in microbial diversity and genomic features. Food Res. Int. 186:114318. 10.1016/j.foodres.2024.114318 38729711

[B38] Sharafi H. Emamjomeh A. Hosseini A. Khaneghah A. M. Moradi M. (2024). Global prevalence and concentration of aflatoxins in meat and edible offal: a systematic review and meta-analysis. J. Food Composit. Anal. 135:106644. 10.1016/j.jfca.2024.106644

[B39] Taormina P. J. Sofos J. N. (2014). “Low-water activity meat products,” in The Microbiological Safety of Low Water Activity Foods and Spices, eds. J. B. Gurtler, M. P. Doyle, and J. L. Kornacki (New York, NY: Springer New York), 127–164.

[B40] Teuteberg V. Kluth I. K. Ploetz M. Krischek C. (2021). Effects of duration and temperature of frozen storage on the quality and food safety characteristics of pork after thawing and after storage under modified atmosphere. Meat Sci. 174:108419. 10.1016/j.meatsci.2020.108419 33418427

[B41] Tlais A. Z. A. Lemos Junior W. J. F. Filannino P. Campanaro S. Gobbetti M. Di Cagno R. (2022). How microbiome composition correlates with biochemical changes during sauerkraut fermentation: a focus on neglected bacterial players and functionalities. Microbiol. Spectr. 10, e00168–e00122. 10.1128/spectrum.00168-22 35699432 PMC9430578

[B42] Vargas D. A. Blandon S. E. Sarasty O. Osorio-Doblado A. M. Miller M. F. Echeverry A. (2022). Shelf-life evaluation of pork loins as influenced by the application of different antimicrobial interventions. Foods 11:213464. 10.3390/foods11213464 36360077 PMC9654175

[B43] Yang P. Zhong G. Yang J. Zhao L. Sun D. Tian Y.. (2022). Metagenomic and metabolomic profiling reveals the correlation between the microbiota and flavor compounds and nutrients in fermented sausages. Food Chem. 375:131645. 10.1016/j.foodchem.2021.131645 34838398

[B44] Zhang Y. Wei J. Yuan Y. Yue T. (2019). Diversity and characterization of spoilage-associated psychrotrophs in food in cold chain. Int. J. Food Microbiol. 290, 86–95. 10.1016/j.ijfoodmicro.2018.09.026 30317110

[B45] Zhou Q. Jiang L. Zhu J. Lu Y. He Q. (2024). The metabolic regulation mechanism of gallic acid on biogenic amines and nitrosamines in reduced-nitrite Chinese fermented sausages: a perspective of metabolomics and metagenomics. Food Chem. 456:139900. 10.1016/j.foodchem.2024.139900 38878551

