# Supplementary material for: Microbial landscape of cooked meat products: evaluating quality and safety in vacuum-packaged sausages using culture-dependent and culture-independent methods over 1 year in a sustainable food chain
Source: Front Microbiol. 2024 Sep 12;15:1457819. doi: 10.3389/fmicb.2024.1457819 (PMC11424441; doi:10.3389/fmicb.2024.1457819)
Supplement: Supplementary file 1 [file Data_Sheet_1.docx]

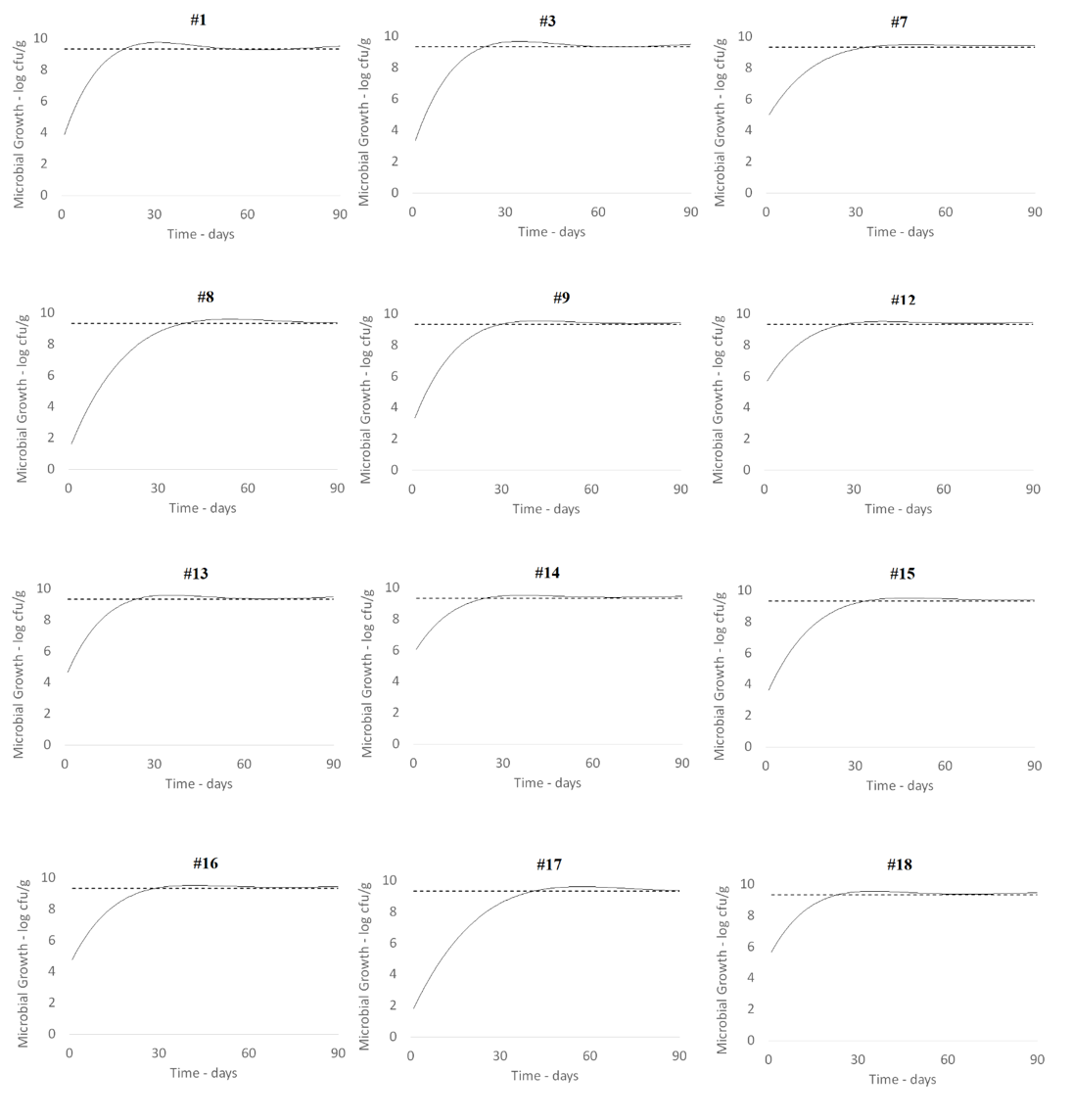


**Figure S1.** Predictive microbial growth curves of natural microbiota growth in vacuum-packaged cooked sausages during summer. The microbial growth predictor (MicroLab_ShelfLife) was used to plot the curves. Temperature profile of the simulation was based on hourly variation during the seasons according to the measurements of the AccuWeather (www.accuweather.com) for 2022 (Figure 1). Latitude and longitude coordinates -22,246 and -43.7031; 22° 14′ 46″ South, 43° 42′ 11″ West (Valença, Rio de Janeiro, Brazil) – humid subtropical climate (Caf) (Köppen-Geiger) (PEEL; FINLAYSON; MCMAHON, 2007). The entrance of the natural microbial community into the stationary phase was considered the borderline of the method.


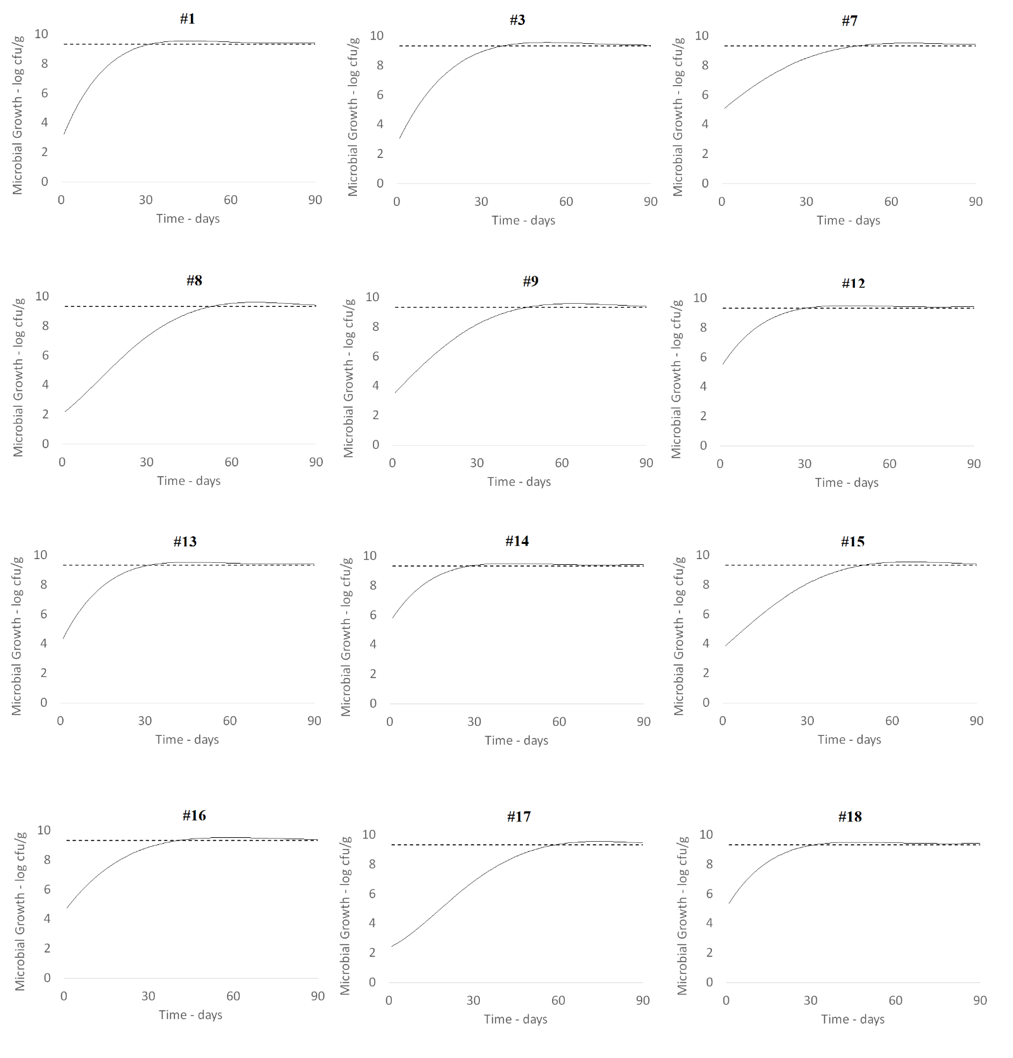


**Figure S2.** Predictive microbial growth curves of natural microbiota growth in vacuum-packaged cooked sausages during autumn. The microbial growth predictor (MicroLab_ShelfLife) was used to plot the curves. Temperature profile of the simulation was based on hourly variation during the seasons according to the measurements of the AccuWeather (www.accuweather.com) for 2022 (Figure 1). Latitude and longitude coordinates -22,246 and -43.7031; 22° 14′ 46″ South, 43° 42′ 11″ West (Valença, Rio de Janeiro, Brazil) – humid subtropical climate (Caf) (Köppen-Geiger) (PEEL; FINLAYSON; MCMAHON, 2007). The entrance of the natural microbial community into the stationary phase was considered the borderline of the method.


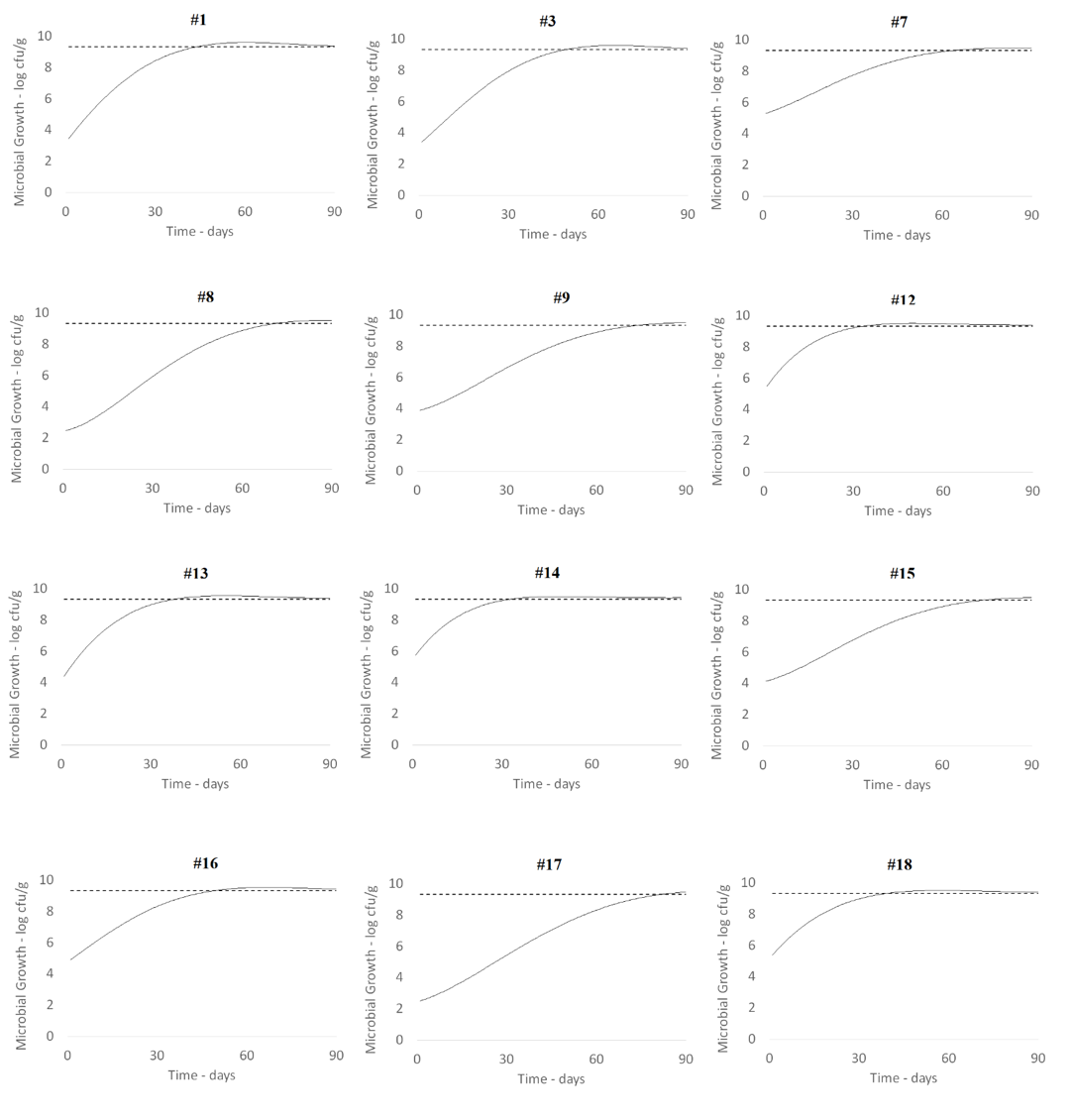


**Figure S3.** Predictive microbial growth curves of natural microbiota growth in vacuum-packaged cooked sausages during winter. The microbial growth predictor (MicroLab_ShelfLife) was used to plot the curves. Temperature profile of the simulation was based on hourly variation during the seasons according to the measurements of the AccuWeather (www.accuweather.com) for 2022 (Figure 1). Latitude and longitude coordinates -22,246 and -43.7031; 22° 14′ 46″ South, 43° 42′ 11″ West (Valença, Rio de Janeiro, Brazil) – humid subtropical climate (Caf) (Köppen-Geiger) (PEEL; FINLAYSON; MCMAHON, 2007). The entrance of the natural microbial community into the stationary phase was considered the borderline of the method.


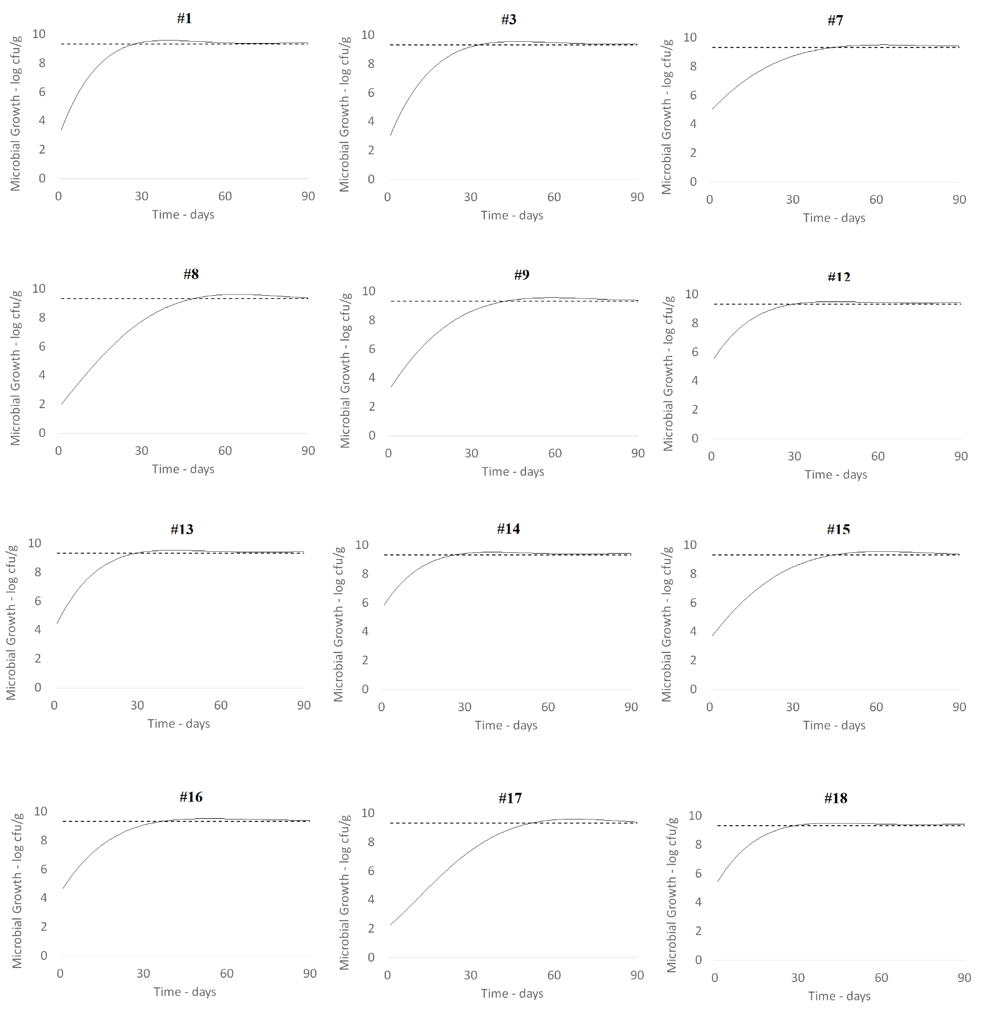


**Figure S4.** Predictive microbial growth curves of natural microbiota growth in vacuum-packaged cooked sausages during spring. The microbial growth predictor (MicroLab_ShelfLife) was used to plot the curves. Temperature profile of the simulation was based on hourly variation during the seasons according to the measurements of the AccuWeather (www.accuweather.com) for 2022 (Figure 1). Latitude and longitude coordinates -22,246 and -43.7031; 22° 14′ 46″ South, 43° 42′ 11″ West (Valença, Rio de Janeiro, Brazil) – humid subtropical climate (Caf) (Köppen-Geiger) (PEEL; FINLAYSON; MCMAHON, 2007). The entrance of the natural microbial community into the stationary phase was considered as the borderline of the method.
